# A hierarchical model for interpersonal verbal communication

**DOI:** 10.1093/scan/nsaa151

**Published:** 2020-11-21

**Authors:** Jing Jiang, Lifen Zheng, Chunming Lu

**Affiliations:** Department of Psychiatry and Behavioral Sciences, Stanford University School of Medicine, Stanford, CA 94305, USA; Wu Tsai Neurosciences Institute, Stanford University, Stanford, CA 94305, USA; Center for Teacher Education Research, Faculty of Education, Beijing Normal University, Beijing 100875, China; State Key Laboratory of Cognitive Neuroscience and Learning, Beijing Normal University, Beijing 100875, China; IDG/McGovern Institute for Brain Research, Beijing Normal University, Beijing 100875, China

**Keywords:** hyperscanning, interpersonal neural synchronization, verbal communication, mutual understanding, interpersonal relationship, predictive coding

## Abstract

The ability to use language makes us human. For decades, researchers have been racking their minds to understand the relation between language and the human brain. Nevertheless, most previous neuroscientific research has investigated this issue from a ‘single-brain’ perspective, thus neglecting the nature of interpersonal communication through language. With the development of modern hyperscanning techniques, researchers have begun probing the neurocognitive processes underlying interpersonal verbal communication and have examined the involvement of interpersonal neural synchronization (INS) in communication. However, in most cases, the neurocognitive processes underlying INS are obscure. To tentatively address this issue, we propose herein a hierarchical model based on the findings from a growing amount of hyperscanning research. We suggest that three levels of neurocognitive processes are primarily involved in interpersonal verbal communication and are closely associated with distinctive patterns of INS. Different levels of these processes modulate each other bidirectionally. Furthermore, we argued that two processes (shared representation and interpersonal predictive coding) might coexist and work together at each level to facilitate successful interpersonal verbal communication. We hope this model will inspire further innovative research in several directions within the fields of social and cognitive neuroscience.

## Introduction

Language distinguishes humans from other non-human animals. Researchers have long been curious about the relation between language and the human brain. Although this issue can be addressed from the neurophysiological or neurological perspective in brain lesion patients (Dronkers *et al.*, 2007), non-invasive imaging of the normal human brain is indispensable. Since the first attempt to conduct research along this line (Petersen *et al.*, 1988), rich evidence has been accumulated during the past three decades. Most of the evidence, however, has been obtained from a ‘single-brain’ or a ‘third-person’ perspective (e.g. for a review, see Redcay and Schilbach, 2019), i.e. when language is processed without a real communication partner. This situation is in conflict with the fact that the purpose of language is to communicate intention and coordinate behaviours between people (Pickering and Garrod, 2004). Thus, it is necessary to study the relation between language and the human brain in an interpersonal communication context.

To meet this call, the previously established modern hyperscanning technique (Montague *et al.*, 2002) and the so-called ‘second-person’ or ‘Two-person neuroscience’ perspective (for reviews, see Hari and Kujala, 2009; Schilbach *et al.*, 2013; Hari *et al.*, 2015; Hasson and Frith, 2016; Redcay and Schilbach, 2019) have been applied to studies of language and the human brain, though to different degrees. These studies show that language communication is associated with a pattern of interpersonal neural synchronization (INS) or neural coupling between partners, i.e. the two time courses of brain activities in two partners covary during the course of communication. The remaining issue, however, is that in most cases, the neurocognitive processes underlying INS are obscure. For instance, although evidence shows that the strength of INS is correlated with the level of mutual understanding, it is not clear how many linguistic and non-linguistic processes are actually involved and how mutual understanding is achieved through these processes when INS is detected.

**Fig. 1.**
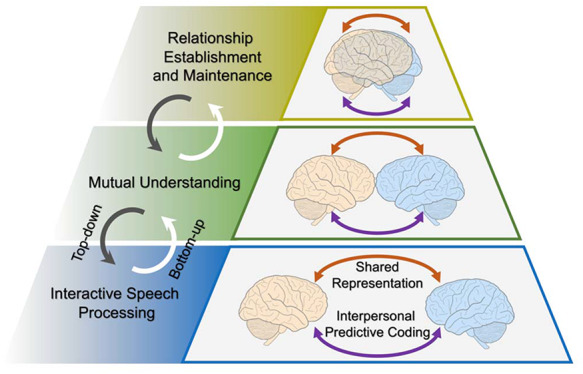
A hierarchical model for interpersonal verbal communication. This model consists of three levels of processes: interactive speech processing, mutual understanding, and relationship establishment and maintenance. At each level, two processes (shared representation and interpersonal predictive coding) work together and are associated with different patterns of interpersonal neural synchronization. Different levels of the model modulate one another bidirectionally in a bottom-up (white arrows) or a top-down manner (grey arrows). The two brains coloured light orange and blue represent communicating partners. The shorter the distance between the two brains, the higher the communication quality and the closer the relationship between communicating partners, and vice versa.

To tentatively address this issue, in this perspective/opinion article, we propose a hierarchical model of interpersonal verbal communication (Figure 1) by reviewing relevant studies that employed a hyperscanning (i.e. concurrent multi-brain scanning during live communication) or pseudo-hyperscanning (i.e. sequential multi-brain scanning during offline communication) technique. The purpose is to facilitate the understanding of the neurocognitive processes underlying interpersonal language communication and INS. Here, we mainly focus on verbal communication, as it is the dominant and most commonly studied mode of language communication compared to non-verbal or sign language communication. The methodological aspects and the non-verbal components of social interaction are not discussed in detail here, as they have been well reviewed elsewhere (for reviews, see De Jaegher and Di Paolo, 2007; Hari and Kujala, 2009; Dumas *et al.*, 2011; Hasson *et al.*, 2012; Wheatley *et al.*, 2012; Schilbach *et al.*, 2013; Babiloni and Astolfi, 2014; Hari *et al.*, 2015; Hasson and Frith, 2016; Gallotti *et al.*, 2017; Bolis and Schilbach, 2018; Minagawa *et al.*, 2018; Shamay-Tsoory *et al.*, 2019; Czeszumski *et al.*, 2020; Köster *et al.*, 2020). However, non-verbal communication will be briefly mentioned when it is relevant. Additionally, our model is formulated from the neurocognitive perspective, i.e. linking different patterns of INS between individuals with different cognitive processes of interpersonal verbal communication, rather than from the computational, pure cognitive or linguistic perspectives. The latter two perspectives have also been discussed elsewhere (Pickering and Garrod, 2004 2013; Hasson *et al.*, 2012; Fusaroli *et al.*, 2014; Friston and Frith, 2015a 2015b; Pickering and Gambi, 2018; Friston *et al.*, 2020). Finally, we conclude this article by highlighting some inspiring future directions in the social and cognitive neuroscience fields.

Moreover, INS has been examined in different forms based on specific neuroimaging modalities chosen in previous studies, such as wavelet transform coherence in functional near-infrared spectroscopy (fNIRS) studies, the phase locking value in electroencephalography (EEG) studies and the Pearson correlation in functional magnetic resonance imaging (fMRI) studies. Despite the different forms, we considered them all as reflections of INS, and thus, all were used as evidence of INS in this review.

## The hierarchical structure of the model

### Interactive speech processing

According to evidence from human archaeology, spoken language has existed for at least a hundred thousand years, which is much longer than that of written language (Tattersall, 2010). It is thus generally recognized that there has been sufficient time for the human brain to evolve and adapt to the process of spoken language (Berwick *et al.*, 2013). As the basis of spoken language, speech processing provides an ideal window into the relation between language and the human brain.

During verbal communication, individuals convey information via vocal sounds. The sounds are produced by the speaker and perceived by the listener, both of which involve widely distributed brain regions such as the early auditory cortex (A1+), classic Broca’s area, Wernicke’s area and premotor area (Wilson *et al.*, 2004 2007; Pickering and Garrod, 2013). Almost all previous models on speech processing, however, focus on either speech perception of the listener, e.g. the dual stream model (Hickok and Poeppel, 2007), or speech production of the speaker, e.g. the DIVA model (Tourville and Guenther, 2011) and the WEAVER++ model (Levelt *et al.*, 1999), omitting the interactive nature of verbal communication.

To address this issue, recent fMRI-based pseudo-hyperscanning studies have been conducted. The evidence has indicated that a distinctive pattern of INS is associated with the interactive speech processing between the speaker and the listener. For instance, Stephens *et al.* (2010) asked a speaker to narrate a personal story in the scanner while the brain activity of the speaker was recorded. Then, the audio recording of the story was played back to the listeners while their brain activities were measured in sequence. General linear model analysis was conducted to assess the relationship between time courses of brain activities in the same brain areas in different participants. The results showed that the brain activity of the speaker during speech production was synchronized with that of the listeners during speech perception in widespread homologous brain areas.

However, the INS identified in this study may arise from the fact that the speaker hears the same vocal sounds during speech production as the listener hears during speech perception. To address this limitation, Liu *et al.* (2020) employed a similar paradigm to that used by Stephens *et al.* (2010), but specifically examined INS between the brain areas related to articulation in the speaker and those related to auditory perception in the listener. The authors found that the brain activities of the listeners in the auditory temporal cortex, including the A1+, middle temporal gyrus (MTG) and superior temporal gyrus/sulcus (STG/STS), were selectively synchronized with that of the speaker in the articulatory motor cortex (i.e. the larynx/phonation cortex) when the listener perceived intelligible speech produced by the speaker. This effect was still valid after controlling the brain activity of the speaker in the auditory cortex and that in the articulatory motor cortex of the listeners (Liu *et al.*, 2020). Moreover, significant INS was found between the A1+ of the listeners and the articulatory motor cortex of the speaker when listening to unintelligible foreign speech (Liu *et al.*, 2020) [notably, this effect was not found between the homologous brain areas in Stephens *et al.* (2010)]. Together, these findings suggest that during interactive speech processing, two types of INS might arise. One type is associated with a common external input such as the vocal sound, which will result in INS between the same homologous brain areas of interacting partners. The other type is associated with interactive linguistic processes such as that between the production and perception of the vocal sound. The latter will result in INS between both the same and different brain areas of interacting partners.

Additionally, previous single-brain evidence has indicated that visual inputs may facilitate or interfere with auditory processing (McGurk and MacDonald, 1976; Skipper *et al.*, 2007). Jiang *et al.* (2012) provided initial evidence to extend this effect from a single-brain situation to a dual-brain situation. Specifically, Jiang *et al.* (2012) had two individuals freely communicate either face-to-face or back-to-back. No scripts were given to the participants for recitation or rehearsal; participants were only given a piece of news about a hot topic that they were to discuss as they would do in daily life. The fNIRS-based hyperscanning was used to simultaneously record brain activities from the two partners. Meanwhile, the entire experiment was video-recorded. The recorded videos were further coded to obtain information on communication behaviours. This information was then linked to INS to understand the underlying cognitive processes of INS. The results showed a significant INS increase in the left inferior frontal gyrus (IFG) between partners during a face-to-face dialogue but not during a back-to-back dialogue, a face-to-face monologue or a back-to-back monologue. Further INS-behaviour linking analyses showed that INS in the left IFG during the face-to-face dialogue was mainly contributed by audiovisual information integration (Jiang *et al.*, 2012). Other fNIRS-based hyperscanning studies confirmed the involvement of the left IFG during face-to-face verbal communication (Osaka *et al.*, 2015). They additionally identified that the right IFG was more closely associated with non-verbal communication compared to verbal communication (Saito *et al.*, 2010; Osaka *et al.*, 2015; Koike *et al.*, 2016). Together, these findings suggest that the INS in the left IFG may serve as the neural base for audiovisual integration during successful interpersonal verbal communication.

### Mutual understanding

By decoding the semantic, conceptual and/or syntactic information that is embedded in the phonological and/or visual signals following interactive speech processing, communicating partners are mutually understood. Although it is obvious that mutual understanding depends on interactive speech processing, it remains unclear whether mutual understanding itself is associated with a distinctive pattern of INS relative to interpersonal speech processing in an interpersonal communication context.

Recently, an increasing number of studies have offered supportive evidence that INS in specific high-order brain areas between communicating partners may serve as the neural base for mutual understanding. For instance, during verbal communication, INS occurs not only in the lower-order linguistic areas but also in a set of higher-order linguistic and extralinguistic areas such as the MTG and STG/STS, temporoparietal junction (TPJ), precuneus, IFG, insula, premotor area, medial prefrontal cortex and dorsolateral prefrontal cortex of the speaker and that of the listener (Stephens *et al.*, 2010; Silbert *et al.*, 2014). Additionally, there is a strong positive correlation between the spatial extent of significant speaker–listener INS and the level of mutual understanding (Stephens *et al.*, 2010). Interestingly, this correlation was found in INS of higher-order brain areas such as the STG/STS rather than in low-order brain areas such as the A1+ (Stephens *et al.*, 2010; Liu *et al.*, 2020). Moreover, INS in higher-order brain areas is not present when listening to unintelligible foreign speech (Stephens *et al.*, 2010; Liu *et al.*, 2017 2020).

Most interestingly, the process of obtaining a mutual understanding can be distinguished from the interactive speech processing based not only on the spatial pattern of INS but also on the temporal pattern of INS, i.e. a temporal-spatial gradient pattern. Specifically, Stephens *et al.* (2010) found that the speaker–listener INS was shown in the A1+ when the time courses of the brain activity of the speaker and that of the listener were temporally aligned; INS also occurred in high-order brain areas such as the TPJ, precuneus and striatum when the time course of the brain activity of the listener lagged behind that of the brain activity of the speaker by ∼1–4 s (Stephens *et al.*, 2010). More direct evidence is provided by Liu *et al.* (2020). Specifically, they calculated INS by shifting the time course of the brain activity of the listener in the auditory areas relative to that in the articulatory motor area of the speaker from −6 s (i.e. the brain activity of the listener preceded the brain activity of the speaker) to 6 s (i.e. the brain activity of the listener lagged behind that of the speaker) at an interval of 2 s. The results showed that INS initially occurred in the A1+ when the brain activity of the listener was time-aligned with that of the speaker, then extending to the STG/STS when the time course of listener lagged behind that of the speaker by 2 s. Finally, INS spread to the MTG when the brain activity of the listener lagged behind that of the speaker by 4 s. These findings were corroborated with data obtained using fNIRS-based pseudo-hyperscanning (Liu *et al.*, 2017). That is, the brain activity of the listener in the parietal areas was significantly synchronized with the brain activity of the speaker in the prefrontal areas with a 5-s time lag for the listener. These findings clearly demonstrated a distinctive neurocognitive process of mutual understanding other than pure auditory speech processing.

In addition, a shared representation of syntax is necessary for successful decoding of semantic information and then achieving a mutual understanding. Previously, robust behavioural evidence showed that individuals tend to use the same syntax as one another during communication (Branigan *et al.*, 2000; Lu *et al.*, 2001; Cai *et al.*, 2012). Recently, Liu *et al.* (2019) provided fNIRS-based hyperscanning evidence for this effect. They had two individuals take turns producing sentences. The communicating partners either employed the same or different syntax in their utterances. The results showed that the use of the same syntax between partners was accompanied by a significantly greater INS in the right posterior STG/STS than what accompanied the use of different syntax. Moreover, INS in this region is significantly correlated with the quality of communication. Therefore, this study further supports the proposition that both shared semantic and syntactic representations are associated with distinctive patterns of INS that are different from those associated with interactive speech processing.

### Relationship establishment and maintenance

Humans are usually organized into different types of interpersonal relationships. The relationships can either be inherent kinship, such as that of parent–child and siblings, or emergent relationships through communication, such as that of teacher–student, friends and romantic couples. Previous evidence has indicated that interpersonal relationships usually emerge when group members act jointly or contingently with each other (Marsh *et al.*, 2009; Algoe, 2019), during which turn-taking plays a key role (Pickering and Garrod, 2004; Wilson and Wilson, 2005). Thus, turn-based interpersonal communication is suggested to be a prerequisite for (Diamond, 2003) and effective in triggering the neurocognitive signatures of interpersonal relationships (Winterheld *et al.*, 2013; Algoe *et al.*, 2017). Even for an inherent kinship such as parent–child relationship, interpersonal communication also plays a supportive role (Eisenberg *et al.*, 1998 2007).

To better understand the role of interpersonal verbal communication in establishing and maintaining interpersonal relationships, the effect of both role assignment and inherent kinships should be excluded. Jiang *et al.* (2015) examined the question of how a leader emerges from a three-member leadless group discussion task. Before the experiment, no leader or follower role was assigned. Additionally, the three members of each group were not acquainted with one another prior to the experiment. The fNIRS-based hyperscanning was employed to test the neural bases of leader emergence. Most importantly, the overall procedures of the experiment were video recorded. Based on the video data as well as additional behavioural assessments, both leadership and various indexes of communication, such as communication skills and competence, initiation of communications and frequencies of verbal and non-verbal communications, were coded. The results showed that a leader spontaneously emerged from the discussion group. Moreover, the emergence of a leader was accompanied by a stronger INS in the leader–follower pairs than in the follower–follower pairs in the left TPJ. Importantly, the quality of verbal communication (i.e. the initiation of verbal communication by the leaders) rather than the quantity of verbal communication (i.e. frequencies of verbal communication between leaders and followers) enhanced the INS of the leader–follower pairs. Interestingly, neither quality nor quantity of non-verbal communication contributed significantly to leader emergence. These findings well demonstrated that verbal communication helps individuals establish leadership when the effects of role assignment and inherent kinships were excluded.

A more interesting finding from Jiang *et al.* (2015) is that INS between leaders and followers can successfully distinguish the leader–follower pairs from the follower–follower pairs 23 s after the onset of communication. Although no other studies on verbal communication during leader emergence have confirmed this finding, studies on non-verbal communication have provided some evidence. For instance, Konvalinka *et al.* (2014) examined dual-brain activity as measured by EEG-based hyperscanning during a finger-tapping task. The roles of the leader and the follower were obtained by behavioural analysis, i.e. the follower was the person who adapted to the taping behaviours of the partner. The results showed a stronger frontal alpha-suppression in the leaders than in the followers during both task anticipation and execution stages. This finding indicates that the difference in brain activity between leaders and followers already appeared before the onset of interaction. This difference, however, might reflect the personal characteristics of leaders rather than interpersonal relationships. Thus, more evidence is needed to show whether the distinctive pattern of INS in leader–follower pairs appear before the onset of communication.

Several studies provided clues about the effect of role assignment, though these studies did not test verbal communication. For instance, Sänger *et al.* (2012) assigned the roles of leaders and followers to participants before dyadic guitar play. Delta- and theta-band EEG signals showed significantly higher values in the phase locking index, i.e. the invariance of phases across trials in the time–frequency domain, in the leaders than in the followers even before the onset of play. However, as only one follower was involved in the experiment, it was not possible to compare INS between the leader–follower pairs and the follower–follower pairs. In a subsequent study, Sänger *et al.* (2013) used a similar paradigm to examine the directional INS between leaders and followers in guitar duets. However, in this study, the authors found significantly higher INS in alpha-band EEG signals from the frontal cortex of the leaders to other brain areas of the followers after the onset of play. Thus, it seemed that role assignment alone was not sufficient to induce INS that is specific to interpersonal relationships.

More direct evidence about the role of verbal communication in the establishment of interpersonal relationships comes from a recent study (Zheng *et al.*, 2020). To separate the effect of verbal communication, role assignment and interpersonal relationships, Zheng *et al.* (2020) employed a dyadic resting-state paradigm. Specifically, haemodynamic brain activities were collected from teachers and students using fNIRS-based hyperscanning when no task was conducted, with participants’ eyes closed, both before and after a one-to-one teaching task. The results prior to teaching did not show any significant INS even when the roles of teachers and that of students had been assigned to participants. Additionally, no significant INS was shown after verbal communication when there was no role assignment among participants. However, when both roles were assigned among participants and verbal communication occurred, i.e. after teaching, a significant increase in INS was found between teachers and students. Moreover, INS after teaching was significantly higher than that before teaching. Additionally, teacher–student INS appeared only after a turn-taking mode of teaching but not after a lecturing or video mode of teaching. Behavioural assessment confirmed the creation of teacher–student affiliative bonds. The strength of affiliative bonds after a turn-taking mode of teaching was significantly higher than that after a lecturing mode of teaching and marginally higher than that after a video mode of teaching. Moreover, the increased INS in the resting state was significantly correlated with the strength of teacher–student affiliative bonds after a turn-taking mode of teaching. These findings together suggest that both the role assignment and the reciprocity of verbal communication are necessary for the establishment of the teacher–student relationship.

Additional evidence comes from parent–child verbal communication. A recent study by Nguyen *et al.* (2020) examined mother–child INS during a free verbal communication task using fNIRS-based hyperscanning. The results showed that not only did mother–child verbal communication induce higher INS than communication between random pairs but also that INS was gradually enhanced over the course of verbal communication. Additionally, turn-taking contributed mostly to the enhancement of INS relative to other indexes of verbal communication, such as content relevance or contingency. Even for preverbal children, modes of communication such as mutual gaze and smiling also contribute to INS between the child and the adult (Piazza *et al.*, 2020). These findings suggest that even for inherent kinships, verbal and non-verbal communications, turn-taking in particular, can further strengthen interpersonal relationships.

Taken together, these findings provide supportive evidence for the important role of verbal communication in establishing and maintaining interpersonal relationships when other factors, such as role assignment and inherent kinships, are considered.

## The processes of interpersonal verbal communication at each hierarchical level

One conundrum that puzzles researchers from both in and outside the field of interaction neuroscience is why the brain activities of two individuals become synchronized during interaction when the brains are not hard wired. Two competing hypotheses can be derived from the current literature. One hypothesis is that a shared representation of the same external stimuli, physical environments or internal mental states/processes along the time course leads to similar temporal and frequency patterns of brain activities between individuals. The similarity of brain activities is demonstrated as an increase in INS. This idea is somewhat consistent with an earlier proposition by Hasson *et al.* (2012), i.e. in dynamic social interaction, INS (aka. brain-to-brain coupling in the original paper), akin to a wireless communication system, reflects the transmission of information (e.g. visual, audio, tactile and/or chemical information) generated by another brain through the surrounding shared physical environment.

Here, we further proposed that such a shared representation might be a general process that is not limited to the physical environment or external stimuli. Rather, it also includes the shared representation of internal mental states or processes. In support of this proposition, previous hyperscanning evidence has shown that a shared mental representation of an action (Yun *et al.*, 2012), syntax (Liu *et al.*, 2019), semantics (Nguyen *et al.*, 2019) or concept (Stolk *et al.*, 2014) between two individuals is associated with an increase in INS when the two time courses of brain activities are temporally aligned. Most importantly, this pattern has been detected in the shared representation of interpersonal relationships such as that presented in a teacher–student relationship (Zheng *et al.*, 2020).

This hypothesis, however, does not fully explain the phenomenon of an observed time delay between time courses of brain activities of two partners when the peak INS is detected. Additionally, it is difficult to interpret why INS sometimes occurs between different brain structures with apparently different brain functions in two partners if only a shared representation process underlies INS. To incorporate these considerations, the second hypothesis is that there is probably another process that is able to explain the time-lagged INS as well as the time-aligned INS between different brain structures. Prediction is a potential candidate to explain these time-related phenomena according to predictive coding theory. This theory has been extensively discussed in single-brain studies (Friston, 2012; Kelly *et al.*, 2019). Briefly, individuals constantly predict future inputs (Aitchison and Lengyel, 2017; Friston, 2002; Spratling, 2017). To this end, an internal model about how to automatically respond to external inputs is generated based on past experiences (Nagai, 2019) and will then be dynamically updated based on the comparison between the prediction and the actual inputs (i.e. the prediction errors) (Friston, 2002; Hertz *et al.*, 2017; Koban *et al.*, 2019). For the dual-brain studies, previous literature provided some clues but unfortunately did not provide details (Hari *et al.*, 2015). A recent review similarly proposed that predictive coding might underlie social synchrony (Shamay-Tsoory *et al.*, 2019). However, this review did not distinguish between the two different aforementioned patterns of INS, i.e. INS between time-aligned brain activities *vs* that between time-lagged brain activities, and INS between the same brain structures *vs* that between different brain structures. Additionally, in their proposition, the authors indicated that the target of the prediction is the gap between two individuals rather than the action or mental states/processes of the partner. Finally, the Integrated Theory of Communication proposed by Pickering and Garrod (2013) and Pickering and Gambi (2018) suggests that individuals make predictions based on their language production system, which is somewhat incompatible with some time-lagged INS findings between the same brain areas. Additionally, this theory does not discuss prediction at the neural level.

Here, we propose that interpersonal predictive coding is another potential process functioning in parallel with that of shared representation and is probably specifically associated with time-lagged INS between the same or different brain structures of two individuals. Moreover, we suggest that during language communication, individuals always seek to minimize the differences between them and the partner in such aspects as actions, semantics/syntax, mental states or processes, and neural representations. This will lead to an increase in similarity between individuals and further demonstrate high-level interpersonal synchronization of behaviours, physiological activity and neural activity.

In this review, we focused on interpersonal predictive coding at the neural level. According to the predictive coding model, the processing of current input can be informed by past experience, suggesting a time lag between the prediction and the current input (Fischer and Whitney, 2014; Kok *et al.*, 2017). Correspondingly, the neural activity of prior prediction in one individual will temporally precede that of inputs from another individual, showing a time-lagged INS. There is evidence supporting this proposition. For instance, based on the time-lagged INS in the sensorimotor cortex, one monkey could make an active prediction on the next action of the interacting partners (i.e. another monkey) (Ferrari-Toniolo *et al.*, 2019). For human beings, evidence is accumulated for each of the three levels of our interpersonal verbal communication model. First, at the interactive speech processing level, the listener usually predicts the subsequent speech of speakers 1–3 s prior to hearing the spoken words (Stephens *et al.*, 2010). In a noisy context, the listener can also predict the subsequent speech of the attended speaker 1–3 s prior to hearing the words, resulting in enhanced time-lagged INS between the listener and the attended speaker (Dai *et al.*, 2018). Second, at the mutual understanding level, i.e. when knowledge was successfully transmitted from the teacher to the student, a significant INS was found when teacher’s brain activity in the TPJ preceded that of the students in the anterior temporal cortex by 10 s (Zheng *et al.*, 2018). Most importantly, this period of time lag roughly corresponded to the periods of asking and answering questions between them (Zheng *et al.*, 2018). Finally, at the interpersonal relationship level, the leader’s brain activity in the TPJ successfully predicts that of the followers, showing temporally causal INS (Jiang *et al.*, 2015). No studies, however, have conducted a computational modelling test on the interpersonal predictive coding hypothesis during a live social interaction, not to mention language communication. Thus, many of the aforementioned propositions are still speculative and are awaiting further tests for verification.

With regard to time-lagged INS, a distinction must be made between INS that is associated with delayed linguistic processing and INS that is associated with a prior prediction from one side of verbal communication. For the former type of INS, a typical example is presented by Liu *et al.* (2020). In that study, a temporal-spatial gradient pattern of INS was revealed between brain activity in the articulatory motor cortex of the speaker and that in several brain areas of the listener. Specifically, INS appeared in the A1+ when the brain activity of the listener was temporally aligned with that of the speaker; then, INS appeared in the STG/STS and MTG when the brain activity of the listener lagged behind that of the speaker in these regions by 2 and 4 s, respectively. While this pattern of INS apparently had nothing to do with the listener’s prediction of the speaker, the shared representation of a specific linguistic process between the listeners and the speaker does not seem to work because of the nature of the time-lag of their brain activities. However, previous evidence has indicated that mesoscale speech rhythm (2–8 Hz) is one of the key features of human speech that underlies the construction of intelligible speech, and the oscillations of neuronal signals or fluctuations of the haemodynamic signals in the brains of both the speaker and the listener might have evolutionarily adapted to the mesoscale speech rhythms (Poeppel and Assaneo, 2020). Thus, it is possible that the shared representation of the mesoscale speech rhythms results in the gradient pattern of INS between the speaker and the listener. A similar effect has also been reported previously during an affective information flow between romantic couples (Anders *et al.*, 2011). Therefore, delayed INS in such a case is different from prediction-related INS. On the contrary, it is likely to reflect the shared representation process.

Finally, interpersonal predictive coding and shared representation might coexist during interpersonal verbal communication. For instance, Liu *et al.* (2019) showed that during verbal communication, the representation of syntax is shared between partners and associated with time-aligned INS in the right posterior STG/STS of the two partners. Additionally, they showed a time-lagged INS between the left TPJ of the two partners, and this time-lagged INS seemed to modulate the shared syntax representations; that is, the shared representations of the direct-object structure of syntax were stronger than those of the prepositional-object structure of syntax during face-to-face communication than during back-to-back communication. However, more evidence is needed to further elaborate on these issues. In addition, the predictive coding and shared representation processes might also work in sequence during an effective teaching, i.e. the teacher first makes prediction on the knowledge level of the student, showing a time-lagged INS; then the knowledge is transmitted from the teacher to the student and shared between them, showing a time-aligned INS (Zheng *et al.*, 2018).

## The main characteristics of the model

Leaning on the hyperscanning evidence we discussed earlier, we synthesize here the hypotheses of our hierarchical model of interpersonal verbal communication in three aspects.

### Hierarchical structure in cognition

During verbal communication, individuals transmit semantic, conceptual and/or syntactic information through vocal sounds to achieve mutual understanding. Sufficient and high-quality communication is helpful in creating and maintaining different types of interpersonal relationships. At the cognitive level, therefore, we propose that three levels of processes are primarily involved in the hierarchical model of interpersonal verbal communication; according to the temporal sequences in which they occur, these three levels are interactive speech processing, mutual understanding, and relationship establishment and maintenance. Moreover, the shared representation and the predictive coding processes might coexist and work together at each level of the model to facilitate successful interpersonal verbal communication.

### Hierarchical structure in brain anatomy

Accumulating hyperscanning and pseudo-hyperscanning evidence has shown that INS in widespread brain areas is closely associated with the processes involved in interpersonal verbal communication. According to the findings we discussed earlier, we argue that interpersonal verbal communication involves INS in both lower-order and higher-order brain areas, in both homologues and heterologous brain areas between communicating partners. Specifically, INS in lower-order brain areas such as the A1+ and articulatory motor area may serve for vocal and/or visual information exchange between individuals. INS in higher-order brain areas such as the STG/STS or TPJ may be more strongly associated with mutual understanding and relationship establishment and maintenance.

### Bidirectionality among different levels of the model

Finally, we propose that the seemingly disparate levels involved in interpersonal verbal communication may modulate one another bidirectionally in a bottom-up or a top-down manner. According to the self-other emerging theory (Levinger and Snoek, 1972; Aron *et al.*, 1991; Aron and Aron, 1997), a close relationship can be conceptualized as overlapping selves or including/merging others within the self. Relevant behavioural evidence showed that the closer the relationship between two individuals, the more common characteristics or the more overlapping features were present in cognitive representations of self and other. On the other way around, the stronger the mutual influence was on each other’s self-representation through communication, the more overlap existed between the two individuals, resulting in the closer relationship (Deutsch and Mackesy, 1985; Agnew *et al.*, 1998). Neural evidence from friendship showed that the closer the friendship in a real-world social network, the higher the similarity in brain responses, i.e. INS between people when freely viewing an audiovisual movie (Parkinson *et al.*, 2018). By borrowing this idea, we further proposed that successful interactive speech processing would enhance the quality of mutual understanding and then further increase the closeness of interpersonal relationships. Likewise, the closeness and/or types of relationships are also likely to modulate INS in the other two levels of interpersonal verbal communication through a top-down manner.

As preliminary evidence for bottom-up modulation, the study by Liu *et al.* (2019) showed that face-to-face interaction with eye-contact mode facilitated INS that was associated with shared syntax representation and mutual understanding. Tang *et al.* (2015) also observed that face-to-face interaction enhanced INS, which was related to shared intention for mutual understanding in an ultimatum game. For top-down modulation, Dai *et al.* (2018) identified that linguistic context selectively affects INS-based interactive speech processing in a noisy situation. Evidence from romantic relationships showed that during naturalistic social interaction, although greater INS was found in the gamma-band EEG signal among romantic couples compared to that found among strangers, INS was only related to non-verbal communication (Kinreich *et al.*, 2017).

## Future directions

The ultimate goal of this model is to enhance our understanding of the relation between language and the human brain in the context of interpersonal verbal communication. We hope that this model will inspire important new questions and help to shed new light on existing issues. First, it is worth noting that in the proposed model, each level of processes involved in interpersonal verbal communication is assumed to be associated with a distinct pattern of INS. However, until now, it remains unclear whether INS occurring at each level is a by-product or a neural cause of interpersonal verbal communication. One potential solution to this problem is to apply non-invasive brain stimulation methods, e.g. transcranial direct/alternating current stimulation or transcranial magnetic stimulation, to modulate brain activity and probably INS prior to verbal communication, thereby testing whether any behavioural change in communications can be observed compared to a control condition or a control group. Importantly, a recent study used 6-Hz in-phase transcranial alternating current stimulation to stimulate the left IFG of two individuals simultaneously while the instructors taught a song to a learner (Pan *et al.*, 2020). The authors found significant interpersonal synchronization in body movement as well as enhanced learning outcomes. Although they did not test the relation between INS and the teaching process, it indicates the possibility of testing the relation between INS and interpersonal verbal communication using non-invasive brain stimulation.

Second, it is of great importance to explore the origin of each level in the model ontogenetically. A growing number of hyperscanning studies have examined non-verbal communication (e.g. joint attention) between infants and caregivers (Hoehl and Markova, 2018). However, these studies may only indicate that infants are able to respond to the non-verbal signals of caregivers when they perceive them. Relatively little is known as to whether they are able to conceptualize the utterances of the caregiver to derive the meaning or even the intention behind the behaviours. Until now, only one study has investigated INS during verbal conversation in children and adults (Nguyen *et al.*, 2020). In the future, more studies are required to address whether the emergence of these abilities is tightly associated with INS in brain regions proposed at different levels.

Finally, our model may provide relevant theoretical implications for the understanding of communication disorders such as autism spectrum conditions (ASCs). It is likely that ASCs may not merely be due to disturbed functions in a single brain but also due to a perturbed attunement between communicating brains (Bolis *et al.*, 2017). Some recent studies identified aberrant INS between an individual with ASC and another person without ASC during an interaction (Tanabe *et al.*, 2012; Wang *et al.*, 2020). However, such aberrant INS may not arise from mere dysfunctions in individuals with ASC. Therefore, embracing both sides of communication and considering all kinds of interactions in which the type of communicating partners is an important factor (e.g. ASC–ASC, ASC–typical, typical–typical) is encouraged (Bolis *et al.*, 2017). Moreover, it remains unknown at which level(s) of process(es) aberrant INS could occur during interpersonal verbal communication in real-life contexts. The findings in this regard could facilitate the identification of the aetiology of a social communication disorder, i.e. either at the individual level or the between-individual level and at which aforementioned process(es) or level(s) of interpersonal verbal communication, thus inspiring the development of novel approaches that target treatments not only for behaviours of an individual patient but also for interactions involving both a patient and the communicating partner.
